# Does amputation side influence sprint performances in athletes using running-specific prostheses?

**DOI:** 10.1186/s40064-015-1470-0

**Published:** 2015-11-04

**Authors:** Hiroaki Hobara, Wolfgang Potthast, Yoko Sano, Ralf Müller, Yoshiyuki Kobayashi, Thijs A. Heldoorn, Masaaki Mochimaru

**Affiliations:** National Institute of Advanced Industrial Science and Technology (AIST), Waterfront 3F, 2-3-26, Aomi, Koto-ku, Tokyo, 135-0064 Japan; German Sport University Cologne, Cologne, Germany; ARCUS Clinics, Pforzheim, Germany

**Keywords:** Prosthetic sprinting, Paralympic, Curved track, Regulation

## Abstract

**Background:**

For athletes using running-specific prostheses (RSPs), current Paralympic guidelines for track events are generally based on level of amputation, not side of amputation. Although 200- and 400-m sprint races are performed in a counterclockwise direction, little is known about the effects of amputation side on race performance in athletes with unilateral lower limb amputation. The study aim was to test whether athletes using RSPs on their left side have slower race times than those using RSPs on their right side.

**Findings:**

Athletes with unilateral lower limb amputation (N = 59 in total) participating in elite-level 200-m races were analyzed from publicly available Internet broadcasts. These races included the 2008 Beijing and 2012 London Paralympics, and the International Paralympic Committee Athletics World Championships in 2011 and 2013. For each athlete the official race time and amputation side were determined. There was no significant difference in number of participants and race time between left and right side amputees in T42 men, T44 men, and T44 women.

**Conclusion:**

The results of the present study suggest that sprint performance of athletes using RSPs is not affected by amputation side on a standard 400-m track.

## Background

Recent technical developments in carbon fiber running-specific prostheses (RSPs) have helped amputee athletes to regain the functional capability of running. For athletes using RSPs, current Paralympic guidelines for track events (Tweedy 2010) are generally based on level of amputation, such as T42 (unilateral or bilateral transfemoral), T43 (bilateral transtibial), and T44 (unilateral transtibial), rather than side of amputation.

In 200-m sprint events, races are performed in a counterclockwise direction, beginning on the curve and ending on the home straight. A previous study (Chang and Kram 2007) demonstrated that during sprinting on a curved track, the inner leg consistently generates smaller peak forces than the outer leg, leading to a reduction of maximum performance of the entire locomotive system. Furthermore, several studies have suggested that the ground reaction forces of the RSPs are smaller than those of the intact leg during straight running (Hobara et al. 2013, 2014; Grabowski et al. 2010). Therefore, the goal of this study was to test the hypotheses that athletes using RSPs on their left leg would have slower race times than those using RSPs on their right leg.

## Methods

Male and female athletes with unilateral lower limb amputation (N = 59 in total) participating in elite-level 200-m races were analyzed from publicly available Internet broadcasts. The distribution of participants is shown in Table 1. Every performance at every competition was considered individually. The races included the 2008 Beijing and 2012 London Paralympics, and the International Paralympic Committee Athletics World Championships in 2011 and 2013. A similar approach to analyze publicly available data from sports competitions for research purposes has previously been used in several studies (Salo et al. 2011; Hobara et al. 2015; Dyer et al. 2014). Institutional review board approval was obtained prior to study initiation.

**Table 1:** Number of participants included in this study and results of Chi square test

	Left	Right	Chi square test
T42 Men	7	9	χ^2^ = 0.25, *df* = 1, *p* = 0.62
T44 Men	7	13	χ^2^ = 1.80, *df* = 1, *p* = 0.18
T44 Women	13	10	χ^2^ = 0.39, *df* = 1, *p* = 0.53
Total	27	32	

First, we determined the race times from the official website of the Paralympic movement (http://www.paralympic.org/). Race times of 200-m sprints of tournament finals were collected. Second, we investigated the amputation side in each athlete through publicly available Internet broadcasts and other sources. Finally, all athletes were categorized into four classes (T42 men, T44 men, and T44 women) and side of amputation (left or right). In the case of T42 men, bilateral transfemoral amputees were excluded from analysis. Further, we also excluded athletes who did not use RSPs.

An independent *t* test was performed for race times to determine significant differences between sides of amputation in each event. Further, to determine whether there is a significant difference in the number of participants in each event, the Chi square test was used. Statistical significance was set at *p* < 0.05. All statistical analyses were performed using SPSS version 19 (IBM Corp., Armonk, NY, USA).

## Results

**Fig. 1:**
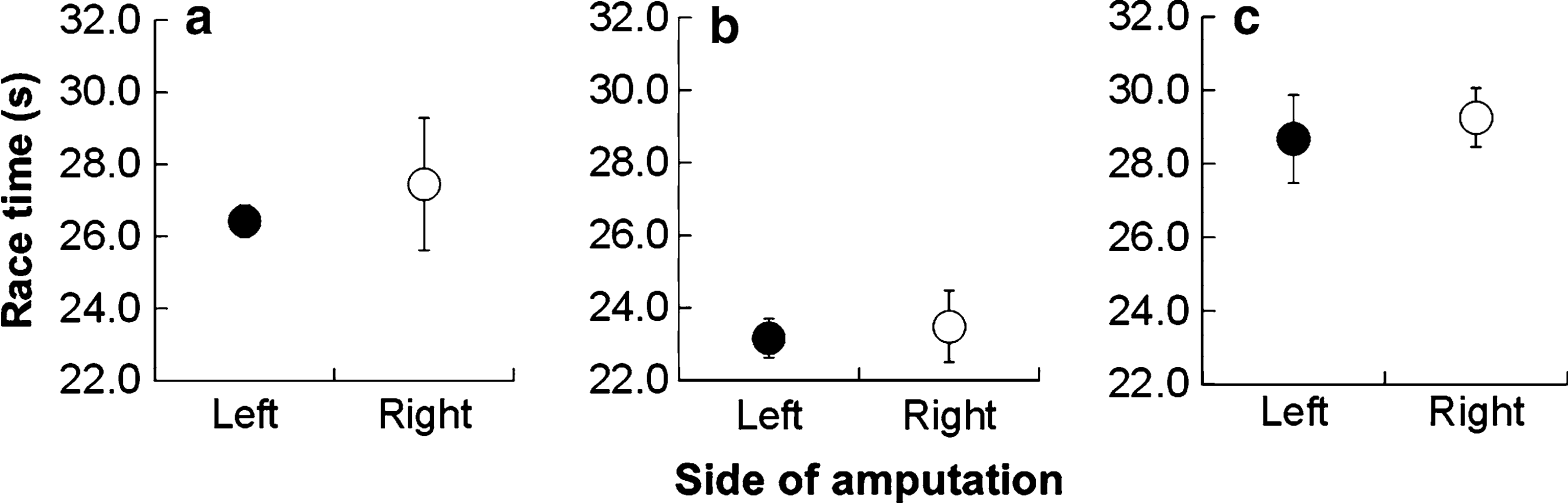
Comparison of race times between left and right side amputations in men’s 200-m T42 (**a**), men’s 200-m T44 (**b**), and women’s 200-m T44 (**c**). *filled* and *unfilled circles* indicate left and right side amputations in each event, respectively

Statistical analysis revealed there were no significant differences in race times between left and right side amputees during any event (Fig. 1). Further, it was found that there were no significant differences in the number of participants between two groups during any event (Table 1).

## Discussion

As shown in Fig. 1, there were no significant differences in race times between left and right side amputees during any event. These results contrast with our initial hypothesis that athletes with left side amputations would have slower race times than those with right side amputations. A possible explanation for the similar race times between left and right side amputees may be the radius of curvature of a standard 400-m track. It has shown that the inside leg consistently generates smaller peak forces compared with the outside leg during curve sprinting (Chang and Kram 2007) and this leads to a reduction of maximum performance on the curved track. However, this previous study investigated circular tracks with radii of 1, 2, 3, 4, and 6 m (Chang and Kram 2007), whereas a standard 400-m track has a radius of 36.5 m (IAAF Track and Field Facilities Manual 2008 Edition). Therefore, the radius of a 400-m track might be too large to observe the same effect as in a previous study　(Chang and Kram 2007).

Current Paralympic sports guidelines (Tweedy 2010) are based on level of amputation, such as unilateral transfemoral amputation (T42) and bilateral (T43) and unilateral (T44) transtibial amputation, not on the side of amputation. Despite the fact that 200- and 400-m sprint races are completed in a counterclockwise direction regardless of amputation side, only a few studies have investigated the effect of amputation side on race performance in athletes using RSPs. As shown in Fig. 1, there were no clear relationships between amputation side and race performance in unilateral amputees using RSPs. Further, there were no significant differences in the number of participants between two groups during any event (Table 1), indicating that there would not be any inherent bias against amputation side during a race. Hence, the results of the current study suggest that amputation side is a factor that does not need to be taken into consideration to ensure fairness in 200- and 400-m sprint events.

There are some limitations to this study. First, we only investigated athletes with unilateral amputation who participated in elite-level competitions, such as the Paralympics and the International Paralympic Committee Athletics World Championship. Thus, caution needs to be taken regarding interpretation and generalization of these findings toward novice and non-expert level athletes. Second, our results are based on retrospective, not experimental, observations. A recent study (Funken et al. 2014) demonstrated that one left sided unilateral transfemoral amputee can run faster in a clockwise direction than in a counterclockwise direction on a curved track due to the asymmetric kinetics. Further, in the present study, stump length in each class and prosthetic knee joint used in Men T42 may vary within each class of amputees. In addition, bilateral differences in strength, elasticity, leg length and balance control in unilateral amputees may induce excessive fatigue in sprinting on a curved track which might cause uneven energy consumption between the legs. Since these factors may affect 200-m sprint performance, further research is required on whether and how sprint performance on a curved track can be affected by amputation side.

## Conclusion

In this study, we tested the hypotheses that athletes using RSPs on their left leg have slower race times than those using RSPs on their right leg. The results of the present study suggest that (1) 200-m sprint performance on a standard 400-m track in athletes using RSPs is not affected by amputation side, and (2) there are no inherent bias against amputation side in athletes using RSPs.
